# Evolutionary optimization of compact dielectric lens for farfield sub-wavelength imaging

**DOI:** 10.1038/srep10083

**Published:** 2015-05-28

**Authors:** Jingjing Zhang

**Affiliations:** 1 Technical University of Denmark, Department of Photonics Engineering - DTU Fotonik, DK-2800 Kgs. Lyngby, Denmark

## Abstract

The resolution of conventional optical lenses is limited by diffraction. For decades researchers have made various attempts to beat the diffraction limit and realize subwavelength imaging. Here we present the approach to design modified solid immersion lenses that deliver the subwavelength information of objects into the far field, yielding magnified images. The lens is composed of an isotropic dielectric core and anisotropic or isotropic dielectric matching layers. It is designed by combining a transformation optics forward design with an inverse design scheme, where an evolutionary optimization procedure is applied to find the material parameters for the matching layers. Notably, the total radius of the lens is only 2.5 wavelengths and the resolution can reach λ/6. Compared to previous approaches based on the simple discretized approximation of a coordinate transformation design, our method allows for much more precise recovery of the information of objects, especially for those with asymmetric shapes. It allows for the far-field subwavelength imaging at optical frequencies with compact dielectric devices.

The resolution of optical imaging instruments, including telescopes and microscopes, can be determined by several factors, among which aberration and diffraction are two major limits. Aberrations can in principle be solved by improving the optical quality – using high-end optical systems with multi-element objective lenses, while diffraction is a fundamental limit that constrains the system’s ultimate resolution. In 1873, the German physicist Ernst Abbe discovered that the diffraction limit arises due to the wave nature of light used and is associated with the finite aperture of the optical elements. His study indicated that a microscope could not resolve two objects located closer than *λ*/2*NA*, where *λ* is the wavelength of light and *NA* is the numerical aperture of the imaging lens, defined as the collection angle of light that enters the objective. The information of object features smaller than the diffraction limit, which is carried by evanescent waves that decay exponentially, is not retained in the far field, resulting in an imperfect image.

For more than a century, various attempts have been made to overcome the diffraction limit that hinders the performance of optical microscopy^1^,^2^,^3^,^4^,^5^,^6^,^7^,^8^,^9^,^10^,^11^. As a paradigm shift, Pendry proposed a ‘superlens’ that allows sub-wavelength resolution^3^,^12^. The sub-diffraction-limited imaging is achieved by recovering the evanescent fields close to the object in a slab of metamaterial with negative refractive index. Although a superlens enhances evanescent modes and thus in principle enables their detection in the close proximity of the lens, the evanescent waves still have faded in the far-field and cannot be processed by conventional optical devices placed far from the superlens. The ‘hyperlens’ seeks to solve this problem by transferring the information carried by evanescent fields into propagating waves, using multilayered metal-dielectric alternating metamaterials which have hyperbolic dispersion contours^8^,^9^. The hyperlens gradually reduces the wave vector component normal to the propagation direction and allows the waves to continue propagating after leaving the lens. However, the implementation of both the superlens and the hyperlens concepts often requires the inclusion of metallic structures, which are inherently lossy and narrowband, thereby severely curtailing the subwavelength resolving power of most superlenses and hyperlenses.

Solid immersion lens (SIL) microscopy^13^,^14^ is an alternative method to achieve sub-diffraction-limited imaging. It combines the advantages of conventional microscopy with those of near-field techniques, and has generated increasing interest, particularly in the field of nanophotonics. By increasing the refractive index of the medium surrounding the focal plane it reduces the local wavelength and thus increases the maximum numerical aperture, resulting in an improvement in the spatial resolution. However, the size of the SIL must be large enough so that the image distortion caused by the impedance mismatch is sufficiently small. Recently, a modified SIL has been proposed based on a dielectric core as well as a gradient-refractive-index shell^15^,^16^. This broadband lens magnifies subwavelength details and delivers information into the far field by generating a virtual image. It has been experimentally demonstrated in microwave frequencies^16^ via a finite number of discrete metamaterial layers to approximate the continuous gradient refractive index. Here, we perform a general study on the modified SIL with a matching shell and theoretically demonstrate that this matching layer with spatially variant material parameters play an important role in precisely delivering the subwavelength information into the far field. Interestingly, we find that although the stepwise approximation provides the most straightforward solution to realize the gradient parameters of matching layer, it results in the distinct distortion of the images for objects without geometrical symmetry (such as reflection symmetry, or some degree of rotational symmetry). To increase the quality of imaging, one would have to increase the number of layers, thereby increasing the size of the lens which would be associated with further dissipation of energy. Therefore, an optimized design of the matching layer is vital to realize a compact lens for effective far-field imaging. Here, we address this problem by applying an evolutionary optimization coupled to analytical Mie scattering theory, where constraints are enforced to guarantee the physical feasibility of the targeted lens within reasonable material and fabrication limitations. We find that this lens can be realized with a dielectric core coated by as few as 4 layers of dielectric materials, while the physical extension of the lens is only 2.5 working wavelengths. The small size of the lens allows the dissipation of the energy to be sufficiently small to not compromise the signal-to-noise ratio, i.e. enough light could be captured on the imaging side for further information processing with a conventional microscope. The optimized lens works effectively to recover the information of even asymmetric objects with the dimension of λ/6.

## Results

### Methodology

**Figure 1:**
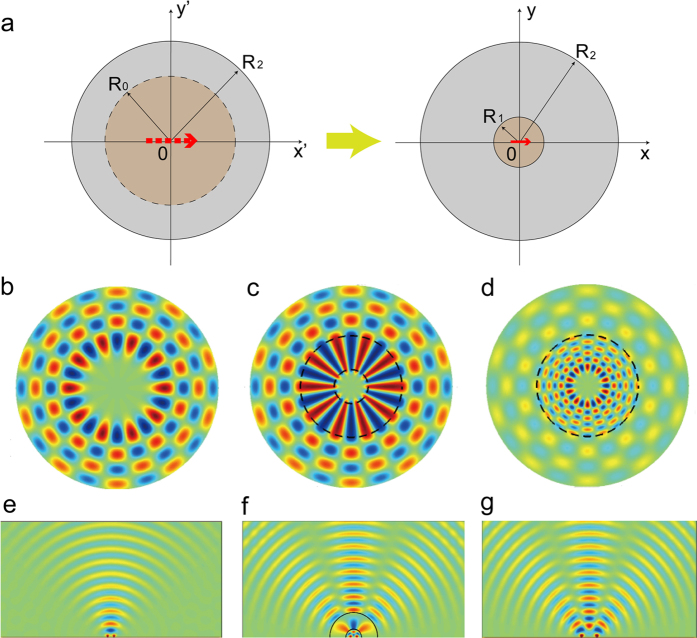
(**a**) The space transformation for designing the modified SIL. (**b**–**d**) Calculated light intensities for n = 10 angular momentum states in free space (**b**), a cylindrical lens composed of a dielectric core (small black dashed circle) as well as a matching layer (large black dashed circle) (**c**), and a large dielectric cylinder (**d**). The magnetic field distributions for two dipoles separated by half of the wavelength in free space (**e**), and immerged in the modified solid lens with magnifying factor 2.5 (**f**). (**g**) The magnetic field distribution for two dipoles separated by 5/4 of the wavelength.

The initial design of this cylindrical SIL is inspired by transformation optics, a methodology that simplifies the modelling of optical devices by warping space to control the trajectories of light rays^17^,^18^. This methodology has proven widespread use in design of novel optical devices and phenomena, including lenses, invisibility cloaks, waveguide tapers, etc.^19^,^20^,^21^,^22^,^23^,^24^, and in study of plasmonic nanostructures^25^,^26^. The strategy is to use coordinate transformations to design landscapes of the optical material parameters that support the desired energy flow and light trajectories through the lens. Here, as shown by Fig. 1(a), by compressing the brown cylindrical region *r *< *R*_0_ (denoted by the dashed circle in the left panel ) to *r *< *R*_1_ (right panel) and correspondingly expanding the gray annulus region *R*_0_ < ρ < *R*_2_ to the region *R*_1_ < ρ < *R*_2_, we obtain a device which magnifies subwavelength features of imaged objects (red solid arrow in the right panel) located in the central core by a factor of *η* = *R*_0_/*R*_1_, creating a magnified virtual image (red dashed arrow in the left panel)^21^. The refractive index of the central core is equal to the magnifying factor *η*, and the coating with anisotropic and spatially variant material profiles works as an impedance matching layer that eliminates the reflection between the core and air^16^. The importance of this matching layer can be appreciated by looking at the scattered angular momentum modes, because the resolution limit of the lens is directly related to the number of cylindrical harmonics involved^8^. Here we compare the high angular momentum states in the free space, the anisotropic SIL, and an isotropic dielectric cylinder of the same size to the lens. Figure 1(b–d) shows the calculated light intensities for *n* = 10 angular momentum states in the three cases respectively. Note how the field decays exponentially in the center of the structures. This suggests that information carried by the high-*m* mode for an object placed in the blank region is lost. In contrast, the anisotropic matching layer allows the high angular momentum state to approach the center without disturbing the intensity distribution outside. In a bare dielectric cylinder with a refractive index of *η*, the high angular momentum state can also penetrate towards the center, but the impedance mismatch causes a severe attenuation of the field intensity outside the cylinder. It is worth pointing out that a dielectric cylinder supports Fabry-Perot resonance modes, and the field distribution in Fig. 1(d) is plotted off the resonance frequencies. At the resonance frequency, the field intensity outside the cylinder can be comparable to or even stronger than that in Fig. 1(c). However, the Fabry-Perot resonances only happen at discretized frequencies, while the SIL in Fig. 1(c) is designed to enhance the transmission for all the angular momentum modes, thereby meeting the requirement of imaging applications.

To show how this modified immersion lens works, we plot in Fig. 1(e,f) the magnetic field distributions for two dipole sources separated by half of the working wavelength. In free space [Panel (e)], the two dipoles cannot be resolved, showing a uniform cylindrical wave pattern. Immerging the dipoles in the modified solid lens with *η* = 2.5 gives rise to a magnified virtual image [Panel (f)]. As a result, the field distribution of the image becomes exactly the same as that from dipoles separated by 5/4 of the wavelength [Fig. 1(g)].

### Anisotropic solid immersion lens and evolutionary algorithm optimization

**Figure 2:**
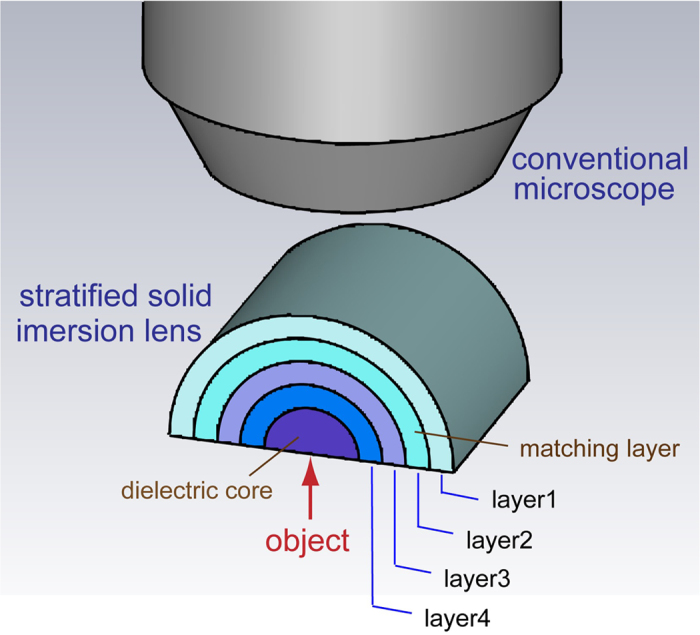
Schematic of the compact SIL with stratified configuration.

**Figure 3:**
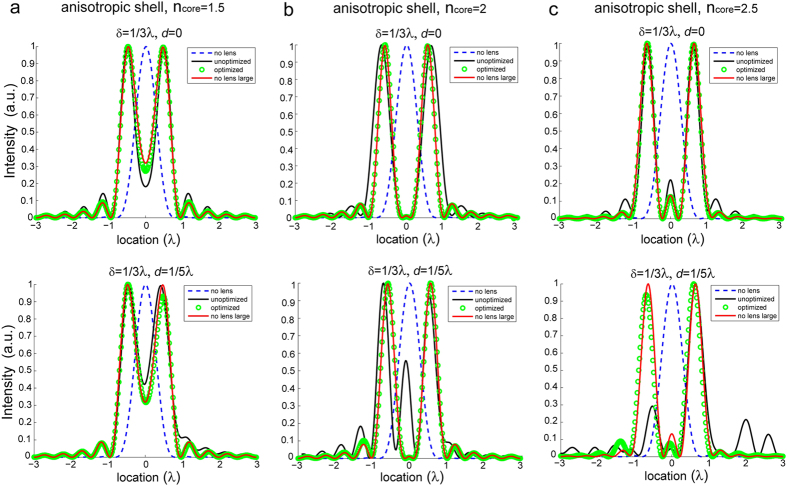
The field intensities of images for two dipole sources separated by δ = *λ*/3, obtained with the unoptimized (black solid curve) and optimized (green circles) SILs in symmetric (top) and asymmetric (bottom) setups. The lens is composed of an anisotropic shell and a dielectric core with *n*_core_ = 1.5 (**a**), *n*_core_ = 2 (**b**), *n*_core_ = 2.5 (**c**). The far-field intensities of dipole sources in free space with separation δ = *λ*/3 (blue dashed line) and δ = *n*_core_*λ*/3 (red solid line) are plotted as a reference.

For transverse magnetic (TM) polarization, the material parameters of the lens can be simplified as
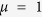

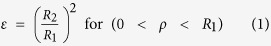

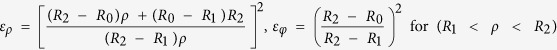
Here, these spatially variant parameters are implemented via a four-layer approximation, and the schematic of the modified SIL with a stratified matching layer is shown in Fig. 2. To verify the performance of the lens we consider different cases corresponding to different magnifying factors *η*. Wave propagation is solved with the aid of a commercially available frequency-domain finite-element method (COMSOL Multiphysics). In particular, we simulate the far-field pattern for two dipoles sources with separation δ = *λ*/3 located in the close proximity to the center of the hemi-cylindrical lens. The near-field intensity of the images can thus be recovered by applying an inverse Fourier transform to the far-field patterns of the harmonic fields (see the method section), as depicted by the black solid curves in Fig. 3. As a reference, we also calculate the field intensities of dipole sources separated by δ = *λ*/3 (blue dashed line), and δ = *ηλ*/3 (red solid line). As the magnifying factor *η* increases from 1.5 to 2.5, the lens always effectively resolves the sources with subwavelength separation although a small deviation from the case of the magnified dipole source separation can be observed. Next, we introduce a shift of the lens with respect to the dipole sources *d* =  *λ*/5. Interestingly, we find that for a lens with a relatively large magnifying factor (*η* = 2 and *η* = 2.5), the image suffers from distinct distortion, which increases with the magnifying factor. This distortion results from the impedance mismatch of the higher-order cylindrical harmonics, and is more prominent as sources are rendered even more asymmetric.

In order to address this problem, we seek and optimized design of matching layers that would yield high-quality imaging. Several topology optimization methods could be used to find optimized landscapes of optical material parameters (including method of moving asymptotes, level-set methods, gradient-based methods, genetic algorithms). Here, we perform an optimization based on evolutionary algorithms (EA) to design matching layers that yield the high-quality imaging, though other approaches could be used as well. Inspired by natural evolution, EAs find the optimized solutions to highly complex nonanalytic problems by repeatedly selecting successful subsequent generations of individuals^27^. These algorithms are well suited for searching open-ended designs with a large number of unknown parameters, and have been applied successfully in various fields of research^28^,^29^,^30^,^31^,^32^,^33^,^34^. Here, we employ the evolutionary optimization method associated with Mie scattering theory to find the permittivity of the matching layer that yields improved imaging performance compared to the one obtained with a simple stepwise approximation. The configuration of the lens under consideration is depicted in Fig. 2. Using the cylindrical wave expansion method, the magnetic field 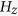 in each layer can be exactly computed and the transmission coefficient 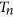 can be derived as a function of the permittivity of each layer (see the method section for more details). Because reconstructing an image is equivalent to retrieving the scattering amplitudes and phase shifts of the various constituent angular momentum modes, an optimum design should ensure that the transmission coefficient 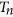 approaches 1 for all cylindrical harmonics. For anisotropic matching layer, this goal can be fulfilled by minimizing the following objective function
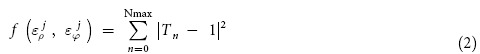
where *j* = 1, 2, 3, 4 (layer number from outer to inner annulus), and *m* is the angular momentum number. Here for simplicity N_max_ is set as 40 in our optimization, because the information carried by very high angular momentum modes is negligible.

The optimized material parameters for three lenses with different magnifying factors (*η* = *n*_core_ = 1.5, 2, and 2.5) are given in Table 1. The image intensities obtained by optimized lenses are depicted by green circles in Fig. 3, where both symmetric and asymmetric cases are considered. We note that the green circles almost overlap the black curves (which correspond to the perfect magnified images) even in asymmetric cases, confirming that optimized lenses effectively yield magnified far-field images.

### Isotropic solid immersion lens and evolutionary algorithm optimization

The implementation of the anisotropic lens requires metamaterials where both the radial and tangential component of the permittivity can be controlled. To simplify the realization of the stratified SIL, the material parameters of the matching layer in Eq. (1) can be further simplified as if *R*_0_ is close to *R*_2_
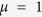

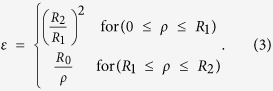


This inhomogeneous isotropic lens works for both transverse electric (TE) and transverse magnetic (TM) polarizations^16^. The gradient parameters can be discretized in the practical realization and have been fabricated by drilling spatially inhomogeneously sized holes in multi-layered dielectric plates^16^.

**Figure 4:**
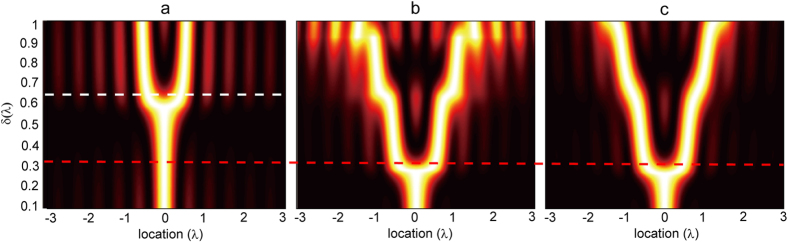
Field intensity versus the source location and the separation between two dipole sources in free space (**a**), covered by unoptimized (**b**) and optimized (**c**) lenses with isotropic matching layers. The magnifying factors *η* of both lenses are 2.5.

Figure 4 shows the field intensities versus the image location and the separation δ for two dipole sources with and without the lens. In free space, as shown in Panel (a), the minimum resolvable distance is around 0.65λ. The resolution of a stratified lens whose parameters are expressed by Eq. (3) (where *R*_1_ = λ, *R*_2_ = 3λ, *R*_0_ = 2.5λ) can be observed in Fig. 4(b), where the dipoles are symmetrically located with respective to the center of the lens. We emphasize that the minimum δ that can be resolved is reduced to 0.3 λ with the lens. However, severe side lobes can be observed especially for large source separations. This is because the discretization of material parameters affects the retrieval of high angular momentum modes, while the contribution of these modes plays a more important role as the source shifts away from the center of the lens.

To solve this problem, here we apply an evolutionary optimization to the simply discretized isotropic parameters. Compared to the anisotropic lens, the isotropic material profile of the matching layer implies a compromise in the performance in retrieving the scattering amplitudes and phase shifts of the constituent angular momentum modes. Hence, it is not possible to achieve an impedance match for all the cylindrical harmonics simultaneously. In this sense, we have to modify the objective function so that the transmission coefficient *T*_*n*_ is uniform for all angular momentum modes, as expressed by
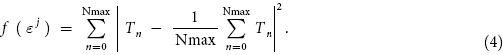


It is worth pointing out that the concentric covers surrounding the core no longer serve as the impedance matching layer, but tend to equal the impedance mismatch of the constituent angular momentum modes (as Eq. (4) aims at equaling *T*_*n*_ for all the cylindrical harmonics involved). In this case, our optimization can still ensure that the images undergo the least distortion, although there is a prize to pay in terms of their reduced magnitude, i.e. relevant to the signal-to-noise ratio. To ensure the feasibility of the material parameters, we set the constraint 0 < ε < 10 in the optimization. The optimized permittivity of each layer is given in Table 2.

The contour plot of the image intensity associated with two symmetrically distributed dipole sources in terms of the image location and the source separation δ is given in Fig. 4(c). We see that the side lobes are dramatically suppressed, and dipole sources with the separation larger than 0.3*λ* can be clearly distinguished.

**Figure 5:**
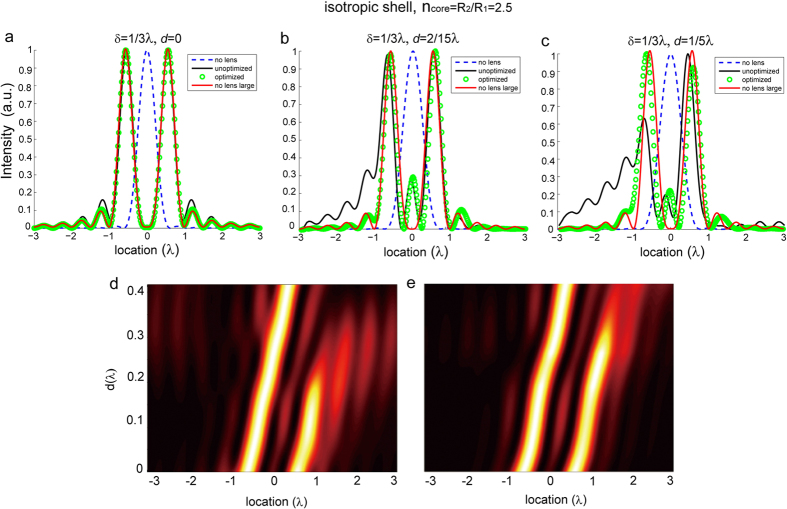
(**a**–**c**) The field intensities of images for two dipole sources separated by δ = *λ*/3, obtained with the unoptimized (black solid curve) and optimized (green circles) SIL. The shift of the lens compared to the center of the source is *d* = 0 (**a**), *d* = 2/15 (**b**), *d* = 1/5 (**c**). The far-field intensities of dipole sources in free space with separation δ = *λ*/3 (blue dashed line) and δ = *n*_core_*λ*/3 (red solid line) are plotted as a reference. (**d**,**e**) The contour plots of the field intensity versus the image location and the shift *d* before (**d**) and after (**e**) optimization. The lens is composed of a dielectric core with *n*_core_ = 2.5 and an isotropic shell.

To investigate the performance of the optimized lens in the imaging of geometrically asymmetric objects, we keep the separation between the dipole sources as λ/3 and introduce a lateral shift of the lens with respect to the center between the two dipoles *d*. The field intensities of images corresponding to *d* = 0, *d* = 2/15λ, and *d* = 1/5λ are given in Fig. 5(a–c), respectively. For comparison, we also calculated the field intensities of images generated by an unoptimized isotropic lens, and of bare dipole sources with small (λ/3) and large (5λ/6) separations. As expected, the images obtained from the isotropic lens design not undergone optimization shows pronounced distortion for relatively large *d*, in contrast to the good performance of optimized lens. The contour plots of the field intensity versus the image location and the shift *d* before and after optimization are shown in Panels (d) and (e), respectively. It further confirms that the unoptimized lens fails to resolve the sources separated by λ/3 when the shift *d* approaches λ/5, while the optimized lens still works well for *d* = λ/5.

**Figure 6:**
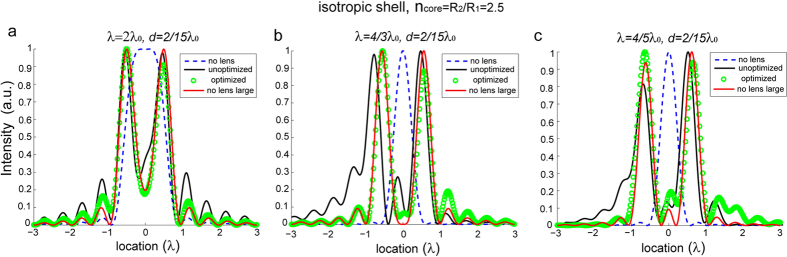
The field intensities of images for two dipole sources separated by δ = *λ*/3, obtained with the unoptimized (black solid curve) and optimized (green circles) SIL at different wavelengths. The far-field intensities of dipole sources in free space with separation δ = *λ*/3 (blue dashed line) and δ = *n*_core_*λ*/3 (red solid line) are plotted as a reference.

To examine the broadband property of this optimized lens, we use the same setup as used in the previous discussions and perform the simulation at three different frequencies, 4λ_0_/5, 4λ_0_/3, and 2λ_0_, where λ_0_ is the frequency at which the optimization is carried out. From the comparison shown in Fig. 6, we see that the optimized lens outperforms the unoptimized one within a bandwidth of 3λ_0_/5. In fact, to achieve an even broader working band, the selected objective functions expressed by Eq. (2) and Eq. (4) can be minimized for multiple frequencies. Naturally, there will be a tradeoff between the bandwidth and the performance of the lens. Besides, the multiple-frequency optimization takes longer time to converge, because the number of generations needed for convergence increases with the number of free variables. With further restrictions, we also anticipate a further reduced magnitude of the image.

I have demonstrated that evolutionary optimization enables the farfield imaging resolution of 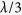 in a broad frequency range, with a compact lens size compared to traditional SIL^14^. This lens can in principle be realized with low loss dielectric materials at any frequency provided that materials with required refractive index are available. It is worth noting that indicated by the transformation optics theory, the resolution of the modified SIL can be further improved by increasing the refractive index of the core and the matching layer. However, high refractive index materials are generally more lossy. For example, at 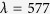 nm, silicon has a permittivity of 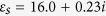
35. Due to the large damping, the energy of light attenuates within ten wavelengths. Therefore, the dimension of the lens must be small to ensure a high quality imaging. To investigate the possibility of achieving even better resolution with the transformation optics design, I extend the constraint to 0 < Re(ε) < 16 and incorporate loss in the evolutionary optimization process (The materials in each matching layer are assumed to have a finite realistic loss). The inner and outer radii of the lens are 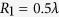 and 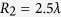, respectively. Table 3 summarizes the permittivity of the matching layers, where parameters derived directly from the transformation approach and corresponding optimized parameters are given.

**Figure 7:**
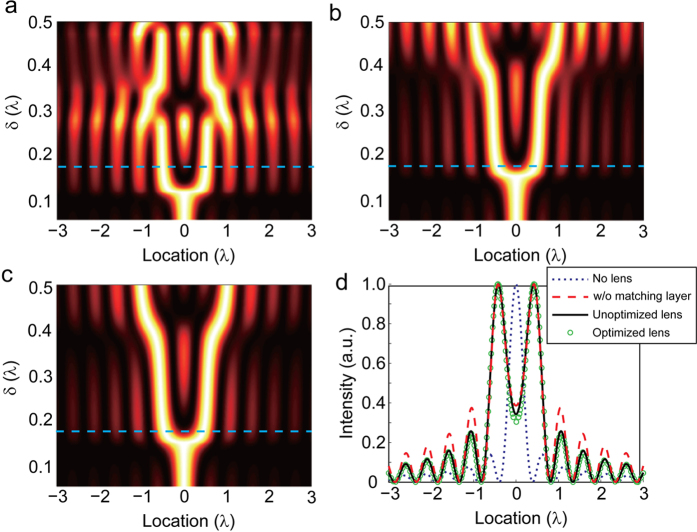
Far-field intensities of the images in terms of the location and the separation between the two point sources in (**a**) a dielectric cylinder without matching layer, (**b**) unoptimized SIL, (**c**) optimized SIL. Panel (**d**) compares the field intensities for two point sources separated by 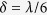 in free space (blue dots), embedded in dielectric cylinder (red dashed line), unoptimized (black line) and optimized (green circles) SILs.

Through numerical simulation, we compare the performance of the optimized lens with that of the unoptimized lens and a dielectric cylinder without matching layer in distinguishing two point sources separated by a subwavelength distance. For the bare dielectric cylinder of such small dimension, strong impedance mismatch is expected, which leads to severe image distortion and large sidelobes, as depicted in Fig. 7(a). On the contrary, the two point sources can be clearly resolved in the transformation modified SIL with and without optimization, while the optimized lens performs better in reducing the sidelobes, as shown in Fig. 7(b). In Fig. 7(d) we can see that the modified SIL can achieve a resolution better than 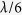.

**Figure 8:**
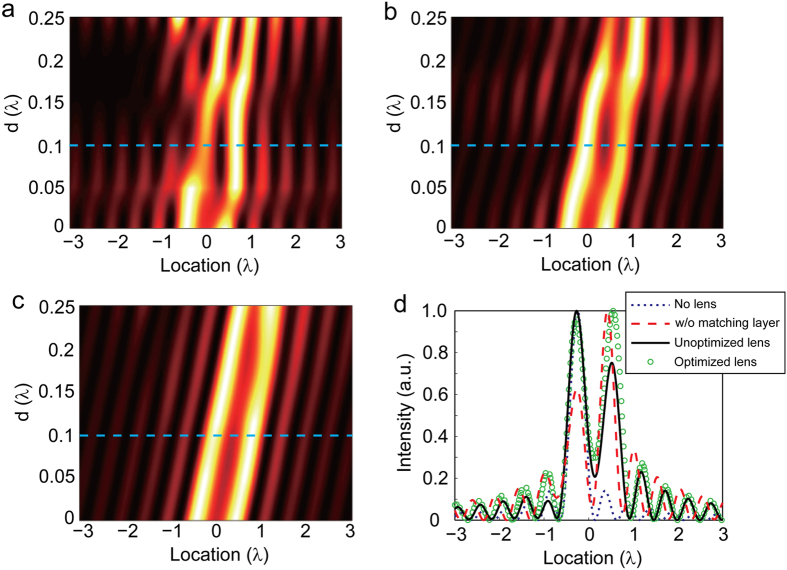
(**a**–**c**) Far field intensity versus the image location and the shift *d* obtained with (**a**) bare dielectric cylinder, (**b**) unoptimized lens, and (**c**) optimized lens. The separation between the two sources is fixed at 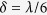 for all the cases. Panel (**d**) compares the quality of the images obtained in different cases when the two sources are shifted from the center of the lens by a distance 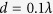

To further investigate the performance of the optimized SIL, I fix the distance between the two sources as 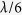 and gradually shift them from the centers of the lenses. As shown in Fig. 8(a), both the cylinder without matching layer and the unoptimized SIL experience a pronounced image distortion when the sources become asymmetric. On the contrary, Fig. 8 (c) show that the lens designed with evolutionary optimization is relatively robust to the position shift, and shows superb performance for a wide range of distances as compared with unoptimized lens.

## Discussion

In conclusion, we present an evolutionary optimization approach to design a SIL composed of an isotropic core and four layers of anisotropic or isotropic matching shells to smoothly deliver the subwavelength information of the object into the far field. Our design can be realized with dielectric materials, and yields better performance compared to previous proposals of gradient refractive index lens designed with coordinate transformation theory. The optimization design approach is not restricted to any specific frequency and can be in principle applied to any frequency range, provided that we set appropriate constraints on the parameters depending on the materials available in the frequency regime of interest to the imaging. The total dimension of the device is considerably smaller as compared with a traditional SIL. Therefore, high dielectric materials, such as silicon in the case of infrared wavelength, can be used to realize this lens without causing significant dissipation of the energy. Although the objective function is evaluated with Mie scattering theory under TM polarized illumination, we find that the optimized isotropic parameters also work for TE polarization. Furthermore, this optimization approach can be extended to the three dimensional case, where a hemisphere solid lens can be fabricated with direct laser writing on dielectric materials.

It is worth noticing that the optimization method presented here can be applied to not only achieve sub-diffraction-limited imaging, but also improve the numerical aperture of a conventional lens. For instance, by allowing the permittivity of the matching layers to vary along the angular direction, it is possible to redirect the scattering of the objects into a small cone. This may help to increase the numerical aperture of the lens in the far-field. Detailed discussions of this problem are beyond the scope of this paper and an extended study on this topic will be performed in our future work.

## Methods

### Genetic Algorithm (GA) Optimization

The evolutionary optimization is implemented via the Global Optimization Toolbox of MATLAB. Here in this case, the initial guess (initial population) from which the optimization starts is the simple discretization of the continuous material parameters given by transformation optics. In the genetic optimization process, different sets of parameters are randomly picked, and a measure of fitness is assigned to each solution, where the ‘fittest’ material parameters (individuals) are kept in each generation. The algorithm incorporates operations analogous to cross-over, random mutations, and natural selection of individuals to create fitter ‘offspring’. This process is repeated until the algorithm terminates when a generation of parameters meets pre-defined convergence criterion.

### Mie scattering theory

I use the Mie scattering theory to calculate the field distribution. When the source is placed inside the device, the magnetic field *H*_*z*_ in each region can be listed as:
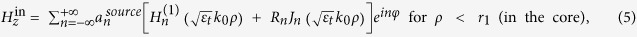

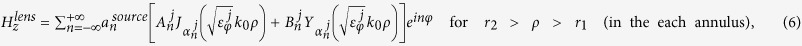

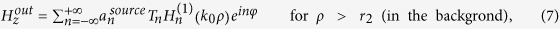
where 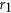 and 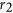 are the inner and outer radii of the device; *j* = 1, 2, 3, 4 (from outer to inner annulus); 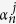 is a constant defined as 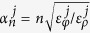; 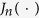, 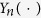, and 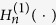 are Bessel function, Neumann function, and Hankel function of the first kind, respectively. The ten boundary equations can be listed, and the transmission coefficient 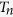 can be derived by solving the above equations:
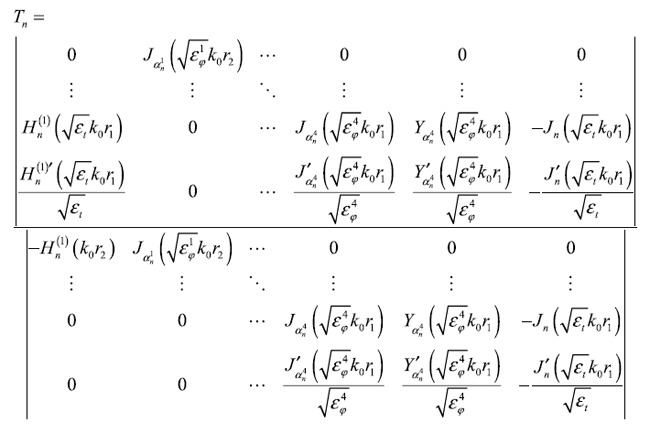
The transmission coefficient as expressed by the above equation is then used for the optimization.

### Numerical simulation and retrieval of images

The far-field pattern obtained from the dipole sources covered by the stratified SIL is simulated with COMSOL Multiphysics 4.4. To recover the image at the source plane, I calculate the field distribution *E*(*x*) at the plane *y* = 0 by performing an inverse Fourier transformation to the far-field electric field using the following normalization
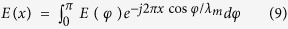
where 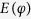 is the angular dependent far-field pattern, and 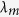 is the working frequency.

## Additional Information

**How to cite this article**: Zhang, J. Evolutionary optimization of compact dielectric lens for farfield sub-wavelength imaging. *Sci. Rep.* **5**, 10083; doi: 10.1038/srep10083 (2015).

## Figures and Tables

**Table 1:** Optimized permittivity for the anisotropic matching layers of the lens with different magnifying factors.

**Magnifying factor**	***η*** = ***n***_**core**_ = **1.5**		***η*** = ***n***_**core**_ = **2**		***η*** = ***n***_**core**_ = **2.5**	
**permittivity**	ε_ρ_	ε_φ_	ε_ρ_	ε_φ_	ε_ρ_	ε_φ_
Layer1	1.0036	1.0567	2.4981	0.3263	8.0492	0.3834
Layer2	1.3735	0.5183	1	0.1840	10	0.3127
Layer3	0.6813	0.2042	1	0.0604	10	0.0527
Layer4	3.1667	0.6817	7.6908	0.6258	10	0.9548

**Table 2:** Optimized permittivity for the isotropic matching layers of the lens with different magnifying factors.

***η*** = ***n***_**core**_ = **2.5**	**Layer 1**	**Layer 2**	**Layer 3**	**Layer 4**
ε	1.3657	4.4688	8.3701	9.4938

**Table 3:** Unoptimized and optimized permittivity of high-resolution SILs designed with high-dielectric materials.

	**Layer 1**	**Layer 2**	**Layer 3**	**Layer 4**	**Silicon Core**
Unoptimized design	1.217+0.016i	1.931+0.025i	3.524+0.045i	8.361+0.107i	16.0+0.23i
Optimized design	4.798+0.061i	5.308+0.068i	13.67+0.174i	15.98+0.204i	16.0+0.23i
